# Sensor-Enhanced Thick Laminated Composite Beams: Manufacturing, Testing, and Numerical Analysis

**DOI:** 10.3390/s24165366

**Published:** 2024-08-20

**Authors:** Mustafa Basaran, Halit Suleyman Turkmen, Mehmet Yildiz

**Affiliations:** 1 Department of Material Science and Engineering, Ayazaga Campus, Istanbul Technical University, Maslak 34469, Istanbul, Türkiye; mustafa.basaran@itu.edu.tr; 2 Engineering and Technology Development, Ford Otomotiv Sanayi. A.S., Sancaktepe 34885, Istanbul, Türkiye; 3 Faculty of Aeronautics and Astronautics, Ayazaga Campus, Istanbul Technical University, Maslak 34469, Istanbul, Türkiye; halit@itu.edu.tr; 4 Faculty of Engineering and Natural Sciences, SU-Kordsa Composite Technologies Center of Excellence and Integrated Manufacturing Technologies Research and Application Center, Sabanci University, Tuzla 34956, Istanbul, Türkiye

**Keywords:** thick laminated composite beams, fiber brag grating (FBG) sensors, structural health monitoring (SHM), finite element analysis (FEA), large deflection, exothermic reaction in composites, three-point bending fatigue test, dynamic and static testing, sensor integration in materials engineering, curing and post-curing analysis, strain

## Abstract

This study investigates the manufacturing, testing, and analysis of ultra-thick laminated polymer matrix composite (PMC) beams with the aim of developing high-performance PMC leaf springs for automotive applications. An innovative aspect of this study is the integration of Fiber Bragg Grating (FBG) sensors and thermocouples (TCs) to monitor residual strain and exothermic reactions in composite structures during curing and post-curing manufacturing cycles. Additionally, the Calibration Coefficients (CCs) are calculated using Strain Gauge measurement results under static three-point bending tests. A major part of the study focuses on developing a properly correlated Finite Element (FE) model with large deflection (LD) effects using geometrical nonlinear analysis (GNA) to understand the deformation behavior of ultra thick composite beam (ComBeam) samples, advancing the understanding of large deformation behavior and filling critical research gaps in composite materials. This model will help assess the internal strain distribution, which is verified by correlating data from FBG sensors, Strain Gauges (SGs), and FE analysis. In addition, this research focuses on the application of FBG sensors in structural health monitoring (SHM) in fatigue tests under three-point bending with the support of load-deflection sensors: a new approach for composites at this scale. This study revealed that the fatigue performance of ComBeam samples drastically decreased with increasing displacement ranges, even at the same maximum level, underscoring the potential of FBG sensors to enhance SHM capabilities linked to smart maintenance.

## 1. Introduction

The legal, environmental, and competition-driven imperatives for reducing CO_2_ emissions, the shift towards electric vehicles, and rapid advancements in material technology have made “lightweighting” technology one of the focal points of the automotive sector. PMCs, developed and utilized in defense and aviation industries, offer a high strength-to-weight ratio, rendering them a technically significant option for the lightweighting studies of automotive applications. Today, thanks to the opportunities provided by advancing technology, PMCs in the automotive sector have expanded beyond non-structural components and are increasingly being employed in load-bearing structural parts.

One of the best examples of the application of structural PMCs in the automotive sector is leaf springs [1]. The leaf springs commonly have a variable cross-section. These non-prismatic structures, known as fully stressed beams, are optimized for material usage [2]. Leaf springs are ideal for structural composite applications due to their beam design, which is tailored for loading conditions limited to vertical (Z-axis) loading and specific loading requirements. This study contributes to the development of composite leaf springs for Heavy Commercial Vehicles (HCVs), which traditionally use steel leaf springs weighing over 200 kg. Composite alternatives offer significant weight reductions, enhancing vehicle damping and driving comfort while featuring high natural frequencies to minimize resonance. Furthermore, the integration of lighter leaf springs in the suspension system reduces the unsprung mass. This diminishes the impact of wheel-originated vibrations on the vehicle body and dynamic load fluctuations on the wheels, maintaining consistent tire–road contact [3]. Consequently, this leads to enhanced comfort and safety.

Despite the limited research on composite leaf springs for HCVs that require thick composite designs between 50 mm and 100 mm due to load requirements, extensive studies have been conducted on Passenger Cars (PCs) and Light Commercial Vehicles (LCVs). These vehicles use PMC leaf springs with a thicknesses less than 50 mm, which are subjected to lower loads compared to usage on HCVs [4,5,6,7,8,9,10,11]. These studies have predominantly focused on understanding the behavior of leaf springs and conducting dynamic modelling. Fatigue behavior is a significant area of study for composite leaf springs due to their exposure to continuously varying dynamic loads. Research simulating actual vehicle usage, such as impact and vibration tests, is crucial to assess the material responses under such dynamic conditions [7,8]. Composite leaf springs are notably significant in the development of structural and dynamic applications for PMCs, mainly due to the challenges posed by fatigue behavior and the complexities of connection techniques [10,11]. Achieving robust and repeatable PMC leaf springs requires careful execution, monitoring, and recording of both the production and validation stages. Critical production parameters include cure temperature, pressure, and time, while validation parameters involve strain, force, temperature, damage evolution and identification, and component life prediction. As stated before, this research focuses on thick PMC beams for designing and manufacturing composite leaf springs, encompassing activities such as determining appropriate manufacturing conditions, identifying and characterizing manufacturing defects, and validating products during fatigue testing using relevant sensor technologies. FE analysis is performed to understand the critical physics observed during the validation stage.

Beyond unique attributes of PMC materials such as high specific strength and enhanced damage tolerance under fatigue loading conditions, they can lend themselves to the creation of smart composite structures by integrating embedded sensor systems during manufacturing [12,13]. This allows the development of advanced composite structures with integrated sensor systems for both manufacturing process monitoring and SHM. SHM provides real-time feedback on load, pressure, and temperature variations in composite structures since it uses intelligent sensors and data analytics for continuous monitoring and immediate fault detection. The SHM system quickly identifies and responds to changes, preventing failures and extending lifespan. Advanced algorithms analyze data to predict performance and guide maintenance [14]. A network of small sensors offers detailed insights, pinpointing damage for timely intervention. Benefits include enhanced safety, cost savings, improved reliability, and data-driven decisions. SHM technologies are mainly based on strain measurements and include Electrical Resistance Strain Gauges, Piezoelectric Sensors, Carbon Nanotube and Graphene-Based Sensors, MEMS Strain Gauges, Wireless Passive Radio Frequency Identification (RFID)-based sensors, and Thermoelastic Stress and Embedded Capacitive Sensors, as well as Optical Fiber Sensors [15]. Among these sensing technologies, RFID strain sensing has attracted notable attention due to its long-transmission-distance capability as well as its semi-active wireless strain sensing features enabled by dual-interrogation-mode RFID technology. However, unlike optical fibers, this technology has some drawbacks, such as susceptibility to environmental conditions (moisture, temperature variation, etc.) and limited range in conductive materials like carbon-fiber composites, especially in passive and semi-passive systems [16,17].

The FBG sensor is one such optical sensor which can be embedded in the PMC structure such that it can be utilized for both processes monitoring and SHM purposes. FBG sensors provide high sensitivity and accuracy in measuring strain and temperature changes. FBG sensors’ ability to be multiplexed along a single optical fiber allows for multiple sensing points with minimal cabling, enhancing efficiency over Piezoelectric and Carbon Nanotube Sensors. Embedded within composite materials without compromising mechanical properties, FBGs also exhibit durability and longevity, enduring harsh conditions better than many alternatives. They deliver real-time data on strain and temperature, crucial for dynamic monitoring and immediate fault detection, with greater accuracy than Thermoelastic Stress and Embedded Capacitive Sensors. Integrated into the manufacturing process, FBGs enable continuous monitoring from production to end-use, offering a comprehensive view of material performance. With low signal attenuation over long distances, FBGs are ideal for large structure monitoring, making them a superior choice for process monitoring and SHM. 

Essentially, FBGs are optical fibers with Bragg gratings inscribed into the core of optical fibers. These gratings reflect specific wavelengths of light while transmitting others. When the fiber undergoes changes in temperature or stress, the spacing of the Bragg gratings changes, causing a shift in the reflected wavelength [18]. The shift in this wavelength (referred to Bragg wavelength, λ_B_) can be precisely measured, allowing for accurate detection of temperature and stress variations as outlined in Equation (1), where Δλ_B_ represents the change in Bragg wavelength, p_e_ is the photoelastic constant (0.22), α is the thermal expansion coefficient (0.55 × 10^−6^/°C), η is the thermo-optic coefficient (8.6 × 10^−6^/°C), and ΔT and ε indicate the temperature change and strain, respectively [19].

(1) $$\frac{\Delta \lambda_{B}}{\lambda_{B}}=\left(1-p_{e}\right)\varepsilon +(\alpha +\eta )\Delta T$$

**Figure 1:**
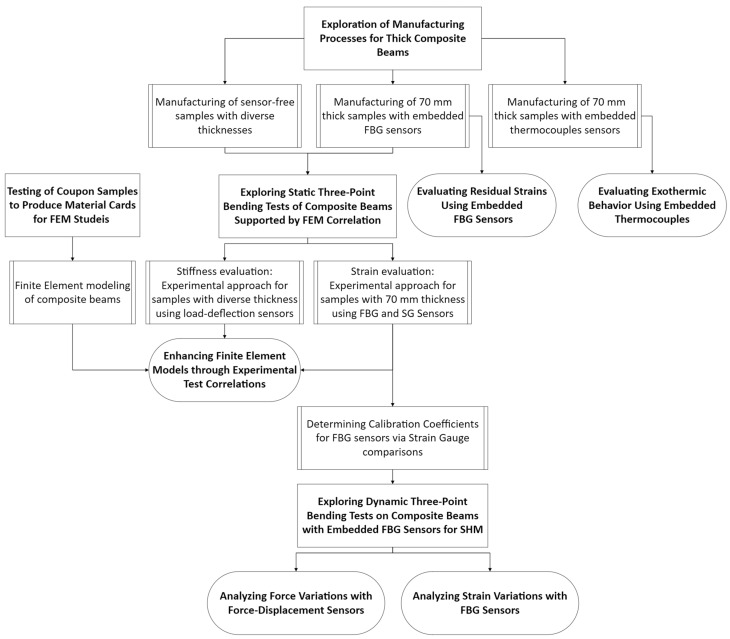
Integrated workflow for the manufacturing, testing, and SHM of large-scale laminated ComBeams using advanced sensor technologies.

This research comprehensively explores the lifecycle of a large-scale laminated ComBeam from its manufacturing stage to physical testing with the help of surface mounted or embedded sensors such as FBGs, SGs, and TCs. Figure 1 presents the detailed workflow of this study. The focus of this study is on the robustness of thick laminated ComBeams, which are crucial for the development of composite leaf springs for HCVs. Unlike prevalent research trends that often concentrate on smaller, coupon-sized specimens, our study emphasizes full-scale composite structures. By expanding this research to include larger structures, size-related factors that significantly influence the mechanical properties of composite materials are addressed and elucidated, as these factors are frequently overlooked in smaller-scale studies.

This study involves fabricating laminated composite samples that imitate the actual dimensions of PMC leaf springs in length and width, while varying thickness to simulate real-world applications more accurately. This approach is crucial for developing a fundamental understanding of the behavior of thick-laminated ComBeams. The study aims to inform basic design principles, enhancing the performance, durability, and safety of modern transportation infrastructure using full-sized composite leaf springs. A key distinction of this study is the use of FBG sensors for process monitoring and SHM. As for process monitoring, in addition to monitoring curing behavior of a thick composite structure, the development of residual strains due to curing a composite structure under a hot compression molding process is investigated in detail through FBG sensors. Additionally, TCs are used to measure temperature profiles at critical stages of manufacturing, providing a comprehensive understanding of curing behavior throughout the production process. In addition to investigations on the manufacturing of thick ComBeams, this research also studies mechanical behavior of laminated ComBeams under both static and dynamic loading conditions. This study extends beyond representative samples to include full-scale prototypes, which are most representative of real-world applications. It is essential to highlight that this work specifically incorporates embedded FBG sensors, SGs, and load-deflection sensors into the experimental setup developed for a three-point bending test, designed to investigate thick composite laminates. This setup is crucial for replicating and understanding conditions comparable to real-life scenarios. Furthermore, numerical studies are conducted with the aim of providing comprehensive insights into the structural behavior of these real-sized laminated coupons, focusing particularly on the outcomes of GNA for thick ComBeams.

This research significantly enhances our understanding of thick laminated ComBeams and underscores the transformative role that FBG sensors play in advancing materials science. It focuses on large-scale, full-sized composite structures, offering insights that are directly applicable to the automotive industry and leading to innovative solutions for transportation and related fields. The study leverages advanced sensor technology integrated into composite materials, representing a crucial step towards ensuring the optimally designed functionality of various structural components in industrial applications.

## 2. Materials and Methods

### 2.1. Material and Manufacturing

Understanding the curing behavior of thick ComBeams with uniform or varying material thicknesses during the compression molding is critically important due to the potential occurrence of an exothermic reaction and non-uniform heat transfer within the material system. FBG sensors are highly effective in measuring temperature, strain, and internal stress distribution. These sensors provide valuable insights into the complex physics of the curing process, including resin gelation, solidification, and shrinkage, which ultimately lead to the development of internal stress [20,21,22,23]. 

**Figure 2:**
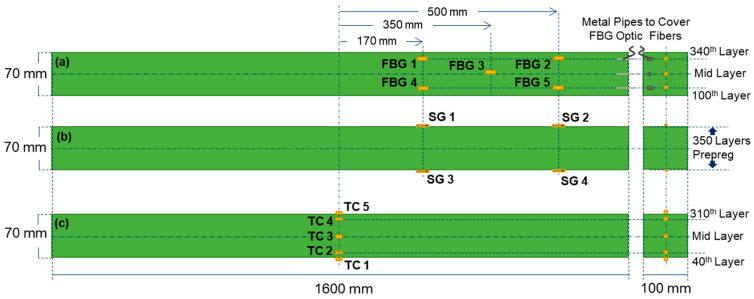
Side view and cross-sectional representation of a laminated ComBeams with varied sensor layout: (**a**) embedded FBG sensors with protective metal pipes near the tip, (**b**) surface-mounted SGs, and (**c**) TCs embedded through the center section.

**Figure 3:**
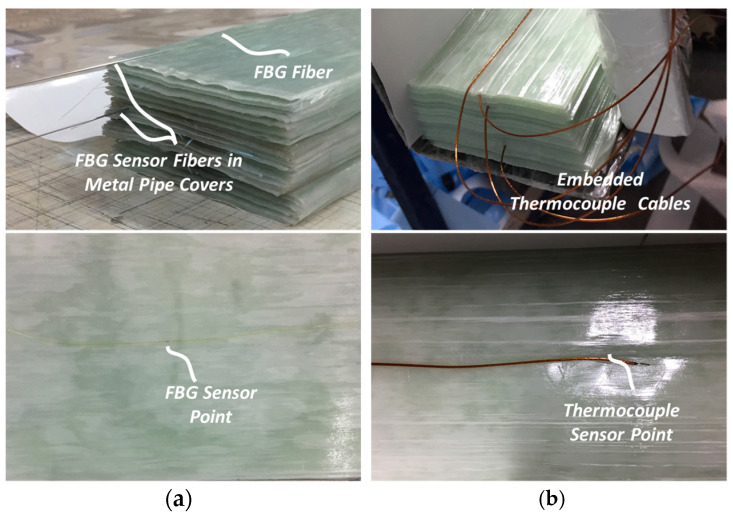
Sensor integration studies in ComBeam fabrication: (**a**) TECHNICA polyimide optical fibers shown with local metal pipe protection at the ends of the laminated ComBeam, including a close-up of an FBG sensor point; (**b**) K-type TC cables embedded within the laminated ComBeams, with a focus on a TC sensor point positioned at the center.

In this study, FBG sensors and TCs were separately integrated into different samples of thick ComBeams with the same dimensions. Single mode polyamide coated FBG sensors with the grating length of 1 mm were purchased from TECHNICA (Atlanta, GA, USA). The ComBeams were produced through using a glass/epoxy 300 Grams per Square Metre (GSM) prepreg, manufactured by Kordsa (Istanbul, Türkiye) with the product name of OM11. The OM11 prepreg is made of unidirectional (UD) E-glass fiber and a hotmelt epoxy resin system with a fiber weight content of 65%. The non-cure ply thickness of the prepreg was measured to be 0.25 mm. For production, the prepregs with a width of 600 mm were cut to strip dimensions of 96 mm in width and 1,595 mm in length using a ZUND Digital Cutter (Zünd Systemtechnik AG., Altstätten, Switzerland). Cut prepregs were hand laid into preforming fixtures to avoid any fiber misalignments along the length of the ComBeams. The total number of laminates was 260, 280, or 350 per ComBeam. All sensors shown in Figure 2 were used for the ComBeam samples with 350 laminates. FBG sensors were strategically embedded between the selected layers during the prepreg layer placement as can be seen in Figure 2a and Figure 3a.

**Figure 4:**
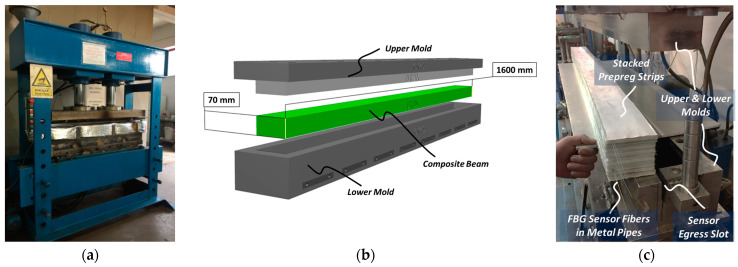
(**a**) Hydraulic press used in ComBeam manufacturing, (**b**) design of thick laminated ComBeam and oil-heated steel molds, and (**c**) mold end adapted for sensor cable retrieval and preformed ComBeam with integrated FBG sensors, each with local metal pipes to protect against mold movement.

It should be noted that the FBG sensors were placed only on the righthand side of the beam, assuming that upon loading the beam experiences the same strain levels on the right- and left-hand sides with respect to the vertical center line passing through the middle of the beam. The sensors FBG 1 and FBG 4 were embedded outside the central clamp area, while the sensors FBG 2 and FBG 5 were positioned near the support points. These boundary locations, which are particularly critical under bending loads, are detailed in Section 2.3. Additionally, sensor FBG 3 was placed on the neutral axis of the beam, equidistant from the other two sensor pair points. The sensors FBG 1 and FBG 2 were placed onto the 340th layer, whereas sensors FBG 4 and FBG 5 were embedded onto the 100th layer, which is dictated by the sensor entry slot of the mold, as detailed in Figure 4b. The TCs (K-type (NiCr/NiAl), acquired from ORDEL (Ankara, Türkiye)) were embedded into middle section of the ComBeams, a critical area for observing exothermic reactions and the development of a thermal gradient during the curing process, as illustrated in Figure 2c and Figure 3b.

The cure kinetics of OM11 resin system was studied in detail using High Pressure Dynamics Scanning Calorimetry (HP-DSC) analysis (Mettler Toledo (Melbourne, Melbourne) HP DSC 2+ system) where the resin samples were heated from 25 to 130 °C with a heating rate of 3 °C per minute. The samples were isothermally held at 130 °C for 30 min to simulate the manufacturing conditions of ComBeams. This process was repeated at different pressure levels: 1, 2, 4, and 6 MPa.

The manufacturing of the beams was conducted using a compression molding method with an oil heated press, as depicted in Figure 4a. After preparing the surface of mold using Axel Xtend-838 mold cleaner (Axel Plastics Research Laboratories, Inc., Monroe, CT, USA), already stacked prepreg strips were placed into the lower mold cavity, as shown in Figure 4b. The mold was specially designed to allow the egress of the FBG sensors and TCs without damage, as seen in Figure 4c. The manufacturing process began with preheating the empty mold to 120 °C, followed by placing the prepreg stacks into the lower mold cavity, closing the upper mold, and consolidating the part under a molding pressure of 125 bars (2000 kN). After molding, the ComBeams were demolded and post-cured in an oven at 110 °C for 10 h to alleviate residual stress within the structure. 

This study produced eight ComBeams in three thicknesses, detailed in Table 1. Samples 1–4 were made to refine production and examine the stiffness and fatigue properties without sensors. Samples 5 and 6 included embedded FBG sensors for monitoring temperature and strain during production and testing. These samples were also used to examine the differential impact of press force on the curing characteristic of samples. Samples 7 and 8 explored exothermic reactions during manufacturing using their embedded TCs.

### 2.2. Experimental Measurements during Manufacturing Processes

**Figure 5:**
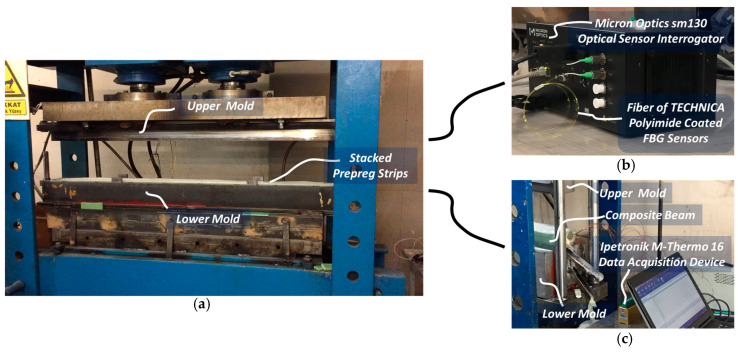
Manufacturing process and data acquisition systems: (**a**) hot compression molding with stacked prepreg strips, (**b**) Micron Optics sm130 retrieving strain and temperature data from FBG sensors, and (**c**) Ipetronik M-Thermo 16 measuring temperature from embedded TCs.

Continuous monitoring of thermal and strain variations was carried out throughout the hot compression molding process, as shown in Figure 5a, and the subsequent post-curing phase. Embedded-FBG-sensor data were captured at a high sampling rate of 100 Hz using the four-channel Micron Optics sm130 optical sensor interrogator, as depicted in Figure 5b. Micron Optics Enlight 1.18.8.0 software (Luna Innovations Inc., Roanoke, VA, USA) was used for the FBG sensor measurements. The Ipetronik (Baden, Germany) M-Thermo 16 data acquisition device (DAQ), shown in Figure 5c, recorded temperature readings at a rate of 1 Hz from the embedded TCs during the manufacturing process and was operated using dedicated Ipetronik 2017 software. Furthermore, data processing was performed using nCode GlyphWorks 2022 analytical software.

### 2.3. Three-Point Bending Tests 

In the PMC domain, it is essential to thoroughly assess mechanical properties under a range of static and dynamic conditions. A considerable amount of research in this field has been realized to investigate the structural properties and performance of PMCs through leveraging FBG sensors, involving strain measurement, residual strain detection and damage monitoring and identification [24,25,26,27,28,29,30,31]. G. Pereira et al. demonstrated the effectiveness of FBG sensors in measuring strain within UD fiber reinforced systems. The strategic positioning of these sensors along the fiber direction within the matrix has been pivotal in validating the correlation between the measured strain and FBG sensor readings [30]. Additionally, various studies in the literature have emphasized the capability of FBG sensors to detect defects in PMC materials by monitoring changes in dynamic strain signals, a technique detailed in references [18,31,32].

Our study contributes to the existing state-of-the-art methods by employing embedded FBG sensors, along with other sensors like SGs and load-deflection sensors, in a specialized testing setup designed for thick composite materials. Specifically, our research employs these integrated sensors in both static and dynamic test scenarios to assess the PMC response to variable loads. 

**Figure 6:**
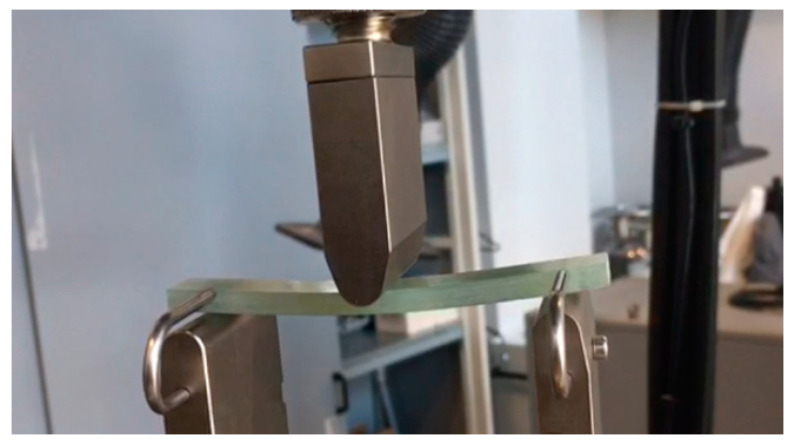
Coupon sample in a three-point bending test on the Instron 5982 test system aligned with ASTM D790 standards [33].

The stiffness of PMC coupons was evaluated through employing a three-point bending test, conforming to American Society for Testing and Materials (ASTM) D790 standards [33]. The coupon samples, dimensioned at 100 mm in length, 15.26 mm in width, and 5.20 mm in thickness (26 layers of UD prepreg), underwent testing utilizing an Instron 5982 universal testing platform (Instron, Norwood, MA, USA) as depicted in Figure 6. The test setup incorporated an 80 mm span for support, with an applied force of 2763 N representing 80% of the ultimate tensile strength of 300 GSM OM11 Prepreg, at a constant frequency of 1 Hz.

**Figure 7:**
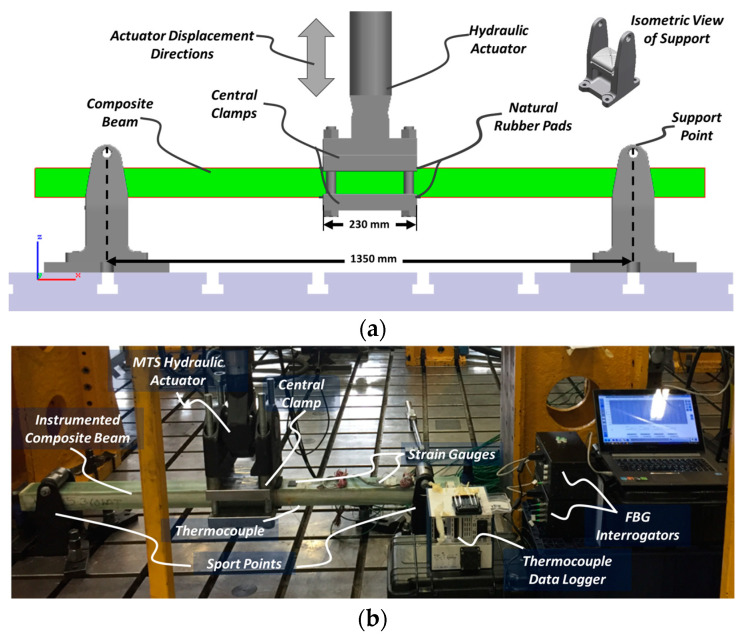
(**a**) Schematic representation of the three-point bending test system for thick composite laminated beams with, and (**b**) photograph of the test system with integrated data collection systems.

Except for ComBeam samples 7 and 8, which are excluded due to the thick TC cables affecting mechanical properties of the ComBeam, the remaining ComBeam samples listed in Table 1 were tested using a custom three-point bending test system, schematically shown in Figure 7a and detailed in Figure 7b. This system simulates operational conditions of leaf springs in HCVs, featuring a 250 kN capacity MTS servo hydraulic actuator (MTS Systems Corp., Eden Prairie, MN, USA) on seismic test plates. The testing protocol involved controlled displacements to precisely measure force and displacement, mirroring the real-world conditions of HCV leaf springs. 

In static stiffness tests, samples underwent a displacement of 75 mm at a frequency of 0.2 Hz using a hydraulic actuator. In addition to stiffness measurements, the FBG sensors were calibrated against the SG results during these static tests. This calibration was crucial for ensuring the accuracy of strain measurements and validating the FBG sensor data, thereby providing a reliable assessment of the stiffness of the ComBeam.

For the dynamic fatigue tests, ComBeam 5 was subjected to cyclic displacements ranging from 35 mm to 70 mm at a frequency of 1 Hz, while ComBeam 6 was tested at 0.5 Hz with displacements ranging from 5 mm to 70 mm. These tests explore the effect of increased displacement on the fatigue life of the material.

SHM was realized using FBG sensors and linear SGs during three-point bending tests. Specifically, ComBeam samples 5 and 6 were instrumented with L2A series linear SGs from Micro Measurements (Raleigh, NC, USA). The experimental setup included a high-accuracy data acquisition system that integrated the FBG sensors with Micron Optics sm130 optical sensor interrogators operating at 25 Hz for precise strain measurement. Additionally, load cell outputs and hydraulic actuator movements were logged at 50 Hz using an MTS Flex 100 Controller, further enhanced by Siemens LMS SCADAS mobile DAQ systems (Siemens Digital Industries Software, Plano, TX, USA) for robust data collection from the SGs. TCs were also placed on the surface of ComBeam samples to monitor thermal effects during fatigue testing, which is crucial for understanding temperature-related changes that could affect the FBG sensor results. The nCode GlyphWorks 2023 software was used to process all of the collected data, including λ_B_, strain, load, and displacement. 

### 2.4. Finite Element Analysis of the Laminated Composite Beams

**Figure 8:**
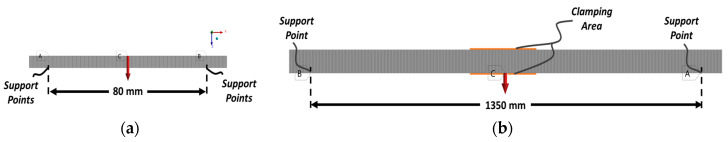
FE models correlating with physical test conditions. Points A and B represent the support areas, while point C indicates the location where the force is applied. (**a**) Depicts a coupon sample in accordance with ASTM D790 standards [33], and (**b**) shows a real-sized ComBeam, both designed to mirror the experimental setup for accurate simulation of the physical tests.

A comprehensive FEA of composite structures was performed, encompassing both coupon samples and real scale laminated ComBeams. The Ansys 2023 software was used to develop the FE models. These models were constructed to accurately reflect the experimental conditions of the physical tests, as illustrated in Figure 8.

In the Finite Element Method (FEM), shell models are developed using material properties from standard coupon tests conducted at ambient conditions, as detailed in Table 2. These properties are integrated to the FE model based on a cured ply thickness of 0.2 mm. The layers are assumed to be perfectly bonded in the FEM. The stiffness analysis incorporates scenarios both with and without GNA, which is crucial for understanding structural behaviors under significant loads and enhancing the accuracy of predictions beyond basic linear elasticity assumptions [34]. 

The computational stiffness results were compared with empirical data from physical experiments to validate the FEM approach, focusing on the effectiveness of the GNA in predicting complex real-world structural behaviors. The FE models for coupon samples and real-scale ComBeams, as detailed in Table 3, were designed to accurately replicate the configurations and thickness variations, thereby enhancing the robustness of the simulations.

**Figure 9:**
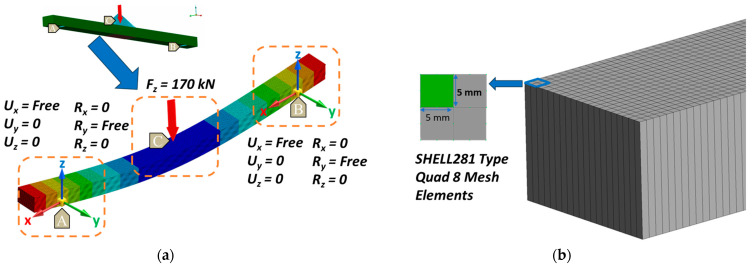
FE model setup for ComBeam samples. (**a**) Demonstrates the boundary conditions aligned with physical test constraints with support points. (**b**) Showcases the model’s precision meshing using 5 mm × 5 mm sized SHELL281 type Quad 8 elements.

Based on the constraints of the physical tests, the boundary conditions for the models were established, as shown in Figure 9a. At the support points, the models were allowed free movement in the x-direction and rotation around the y-axis. However, movements in the y and z directions, as well as rotations in the x and z axes, were restricted to replicate the physical conditions.

Meshing was performed with precision using SHELL281 type Quad 8 mesh elements, as shown in Figure 9b, with the 5 mm × 5 mm mesh sizes. A total of 6400 shell elements were used for the ComBeam FE models (FEM 260, 280, and 350), and 400 elements with 2 mm × 1.9 mm sizes for the coupon model (FEM 26). 

Forces of 1 kN, 100 kN, 115 kN, and 170 kN were applied to the FEM 26, FEM 260, FEM 280, and FEM 350 models, respectively. These magnitudes were chosen to replicate the quasi-static three-point bending test conditions in the experiments. The static test output of strains from the embedded FBG sensors and SGs were validated with the FEA results, which helped in the correlation of the FE models with real experimental data. 

## 3. Results and Discussion

### 3.1. Investigation of Cure and Post-Cure Processes

The results of the HP-DSC studies and the experimental studies during the curing and post-curing processes are presented in this section. The information obtained through FBG sensor and TC measurements in different manufacturing stages is important to clarify material response in thermal and pressure environments. These data allow us to analyze the development of internal stress and thermal variations, both being critical means for quality and reliability assessment for the composite structure. During these processes, the response of the material was analyzed, and the emphasis was to acquire a quantitative estimate of the curing behaviors that provides a better understanding of the residual stresses that persist into the post-cure phase. 

**Figure 10:**
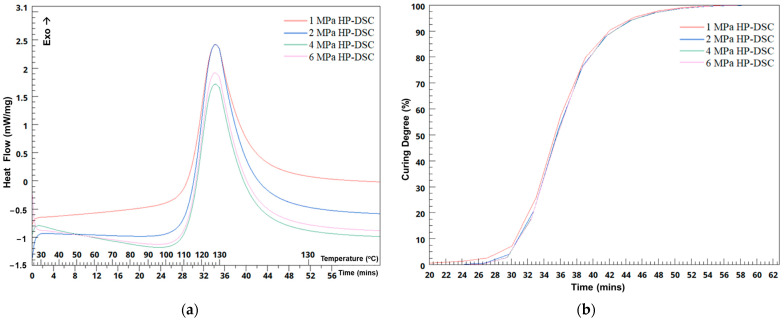
Thermal behavior and curing process of the OM11 resin system under varying pressures via HP-DSC. (**a**) Minor heat flow variations with increasing pressure, indicating a consistent thermal response. (**b**) The average degree of curing remains unaffected by pressure changes, underscoring the stable curing characteristics of the resin across different pressure levels.

The HP-DSC studies, as shown in Figure 10a, indicate minimal pressure dependency on heat flow when pressure varies from 1 MPa to 6 MPa. This aligns with existing research stating that pressure changes do not influence the curing characteristics of uncured epoxy-based prepreg materials [35]. A slight decrease in mean heat flow with increased pressure suggests minor effects on exothermic reaction rates. The material exhibits uniform responses to both heat and pressure, with consistent standard deviation levels across different pressures. Additionally, Figure 10b demonstrates that the degree of curing remains largely unchanged over time under various pressures, confirming that pressure does not significantly impact the curing degree.

As the pressure is incrementally raised from 1 MPa to 6 MPa, the recorded onset temperatures exhibit negligible variation, with values of 112.86 °C, 112.85 °C, 112.46 °C, and 111.30 °C, respectively. This suggests stable curing temperatures despite the changing pressures. However, it is imperative to acknowledge that while the HP-DSC studies indicate minimal impact on the curing behavior within this pressure range, practical manufacturing scenarios, such as the production of full-sized components like ComBeam samples, present a different picture. In these real-world manufacturing settings, factors such as the uniform impregnation of resin within the composite structure, resin flow dynamics, and the presence of voids are notably influenced by applied pressure. This disparity underscores the limitations of HP-DSC in capturing the complexities inherent in practical manufacturing processes, particularly concerning resin voids, their distribution, and resin impregnation. While HP-DSC offers valuable insights into material behavior under controlled laboratory conditions, it fails to encapsulate complexities observed in actual manufacturing environments. This underscores the importance of integrating laboratory research with practical manufacturing experiences to gain a comprehensive understanding of material properties and their processing techniques.

**Figure 11:**
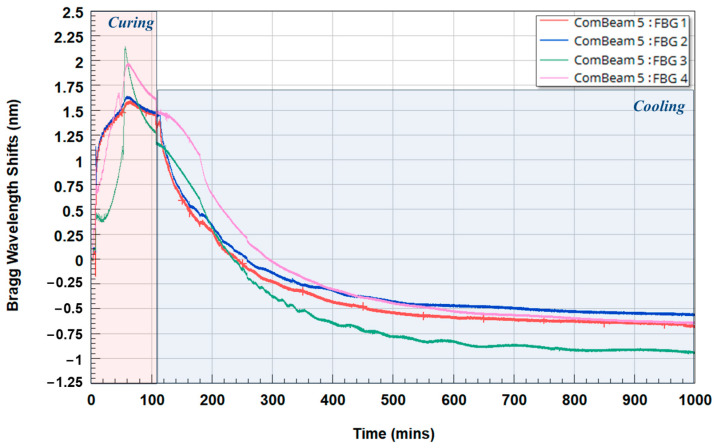
Wavelength variations of four FBG sensors in ComBeam 5 during curing and cooling phases.

Figure 11 shows the Bragg Wavelength Shift (Δλ_B_) of ComBeam 5 from FBG sensors 1 to 4, with sensor positions detailed in Figure 2a, starting from stacked prepreg strips at ambient temperature through the manufacturing and cooling phases until equilibrium with the ambient temperature is reached. The Δλ_B_ (Y-axis) in nanometers indicates strain and/or temperature changes, with time in minutes on the X-axis. This response is noted by a marked significant shift in the wavelength during the curing phase, suggesting both a reaction to the exothermic nature of the chemical reactions taking place within the composite and mechanical stresses applied when the pressure of hydraulic press is increased. The decrease in the curve of the Bragg wavelength after the peak indicates that the exothermic reaction has completed and that there is a relaxation of internal stress. The cooling phase in the graph starts after the mold is opened and continues until equilibrium is established with the ambient temperature. 

Figure 11 shows that at the end of the cooling phase, when the entire structure reaches ambient temperature, the FBG 3 sensor, positioned at the center of the cross-section, experiences the largest negative shift in λ_B_. Since the structure is also at ambient temperature at the beginning of the process, the thermal effect on the shift, as indicated by Equation (1), can be eliminated. This observation on Δλ_B_ suggests that residual stress varies by cross-sectional location, peaking in the central area where the highest level of exothermic temperature rise is observed upon curing. To further elaborate on this result, the Δλ_B_ were assessed to evaluate residual strain in ComBeam 5 and ComBeam 6. This observation is consistent with findings from previous studies [36,37], which emphasize the importance of residual strains as indicators of manufacturing irregularities. A comprehensive analysis of the residual stress at each sensor location, both after curing and post-curing, is presented in Table 4, which lists the residual strain values after curing and during the post-curing phases, along with the percentage change in residual strain which indicates the relaxation of the composite material over time. The values of calibrated residual strain for the FBG sensors have been derived using the CCs calculated in Section 3.2.

Table 4 presents the measurement and calibration analysis of residual strains using FBG sensor readings for ComBeam 5 and ComBeam 6, offering detailed CCs. Initial post-curing measurements show residual strain variations ranging from −469 to −798 µε in ComBeam 5, with FBG 3 recording the highest value. After post-curing, these values shift to −383 to −746 µε, indicating a reduction in internal stresses. The percentage change in remaining strain for sensors in ComBeam 5 ranges from 7% to 21%, with FBG 1 showing the highest decrease. Similarly, in the post-curing phase of ComBeam 6, the residual strain decreases. For instance, FBG 1 exhibits a strain of −577 µε, which decreases to −500 µε, representing a 13% decrease in in-plane residual strain. The percentage change in residual strain for ComBeam 6 ranges from 9% to 18%, suggesting that the composite material gradually relieves internal stresses over time due to the different curing processes. The residual strain values after curing and post-curing phases, along with the percentage change in residual strain, indicate the relaxation of the composite material over time.

**Figure 12:**
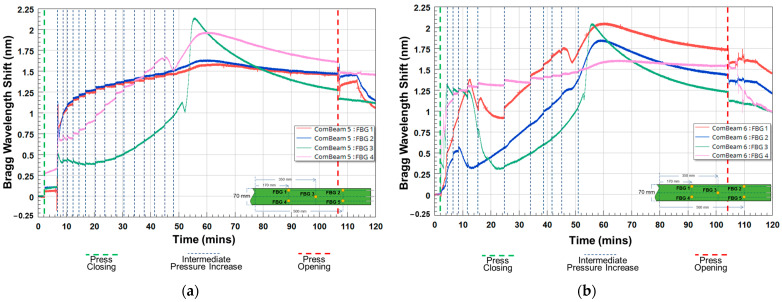
The Δλ_B_ as recorded by FBG sensors during the curing process of (**a**) ComBeam 5 and (**b**) ComBeam 6.

Figure 12 presents two different consolidation procedures and their effect on the recorded FBG sensor data in terms of Δλ_B,_ for ComBeam samples 5 and 6 during the curing stage. Each plot represents the shift for individual sensors (FBG 1 to FBG 4), indicating the response of the material to thermal and mechanical stresses throughout the process. These shifts are directly related to the increase in the applied pressure by the hydraulic press and to the temperature changes during the process (mold heating and exothermic reaction), as well as the possible residual strain formation as a result of the curing reactions. The vertical lines in Figure 12 indicate press movements, including press closing, intermediate pressure increases, and press opening times, all correlated with the timing of the Δλ_B_. The inset figures provide the location of the sensors within the ComBeams. For the curing process of ComBeam 5 (Figure 12a), the force is initially applied at 750 kN and then increased to 2000 kN after 5 min. In contrast, ComBeam 6 (Figure 12b) is directly compressed with 2000 kN without any initial force.

**Figure 13:**
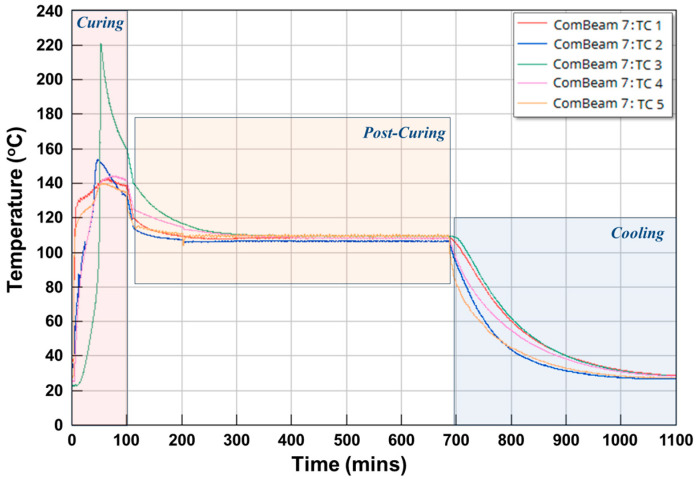
Thermocouple measurements illustrating the temperature variations of ComBeam 7 throughout the manufacturing stages.

In Figure 12a, it is evident that upon closing the press and applying pressure to the composite structure, a small increase in the wavelengths of all FBG sensors was observed. This phenomenon is attributed to the improved contact between the composite layers under pressure, which induces tensile stresses on the FBG sensors. Sensors located closest to the surface of the ComBeam exhibit a rapid increase in wavelength due to the fast transfer of heat from the mold. Among the sensors, FBG 1 and FBG 2 show the fastest initial wavelength increase, followed by FBG 4 and FBG 5. Although the FBG 4 and FBG 5 sensors were near the surface, their positions between inner layers cause a slower heat transfer from the mold, resulting in a more gradual wavelength increase. Positioned at the midsection of the beam structure, sensor FBG 3 experiences the slowest heat transfer from the mold, leading to a relatively slower increase in wavelength. Sensor FBG 3 shows the most significant wavelength change among all sensors because the heat generated by the curing reaction takes longer to dissipate to the mold and surrounding environment, which is corroborated by the TC measurements given in Figure 13. The other sensors, being closer to the mold surface, experience faster heat dissipation of the exothermic reaction by the mold. Assuming that the FBG 1 sensor reaches the mold temperature within approximately 10 min, it can be inferred that the curing process completes around the 110th minute, as the wavelength changes for FBG 1 are consistent between the 10th and 110th minutes. Similar general discussion applies to the ComBeam in Figure 12b, although the wavelength changes patterns of the FBG sensors differ. These variations are likely due to the different consolidation procedures, as stated previously, as well as statistical variations in manufacturing processes. As such, when the pressure is applied and subsequently increased, the embedded FBG sensors adhere or bond more effectively to the composite layers they are embedded in. Due to the gradual application of the consolidation pressure, the resin can flow smoothly at mold temperature without disturbing the positioning of FBG sensors and altering the thermal field in their vicinity. This results in the FBG sensors experiencing a uniform wavelength change within the structure induced by stress and temperature fields. If the pressure is applied rapidly instead of incrementally, the resin attempts to flow quickly towards the lateral surfaces of the composite structure, causing fluctuating changes in the FBG sensors’ wavelengths. 

Temperature measurements from the TCs during the curing, post-curing, and cooling phases of ComBeam 7 are presented in Figure 13. In this figure, the temperature changes of each TC are different due to factors such as the low thermal conductivity coefficient and the exothermic reaction characteristics of the OM11 resin system. Additionally, there is a temperature difference of around 5 °C between the upper and lower molds. Although the molds are set to 130 °C, the ComBeam samples reach over 200 °C due to the heat generated during the exothermic reaction, which is not transferred out of the thick composite structure because of the resin system’s low thermal conductivity coefficient. After the curing phases, temperature homogenization during the post-curing phase and a slow decrease in temperature towards ambient conditions during the cooling phase, can be observed in Figure 13. This temperature profile is important as it represents the internal thermal gradient, which governs the rate and extent of the cross-linking reactions critical to the curing of the matrix. 

**Figure 14:**
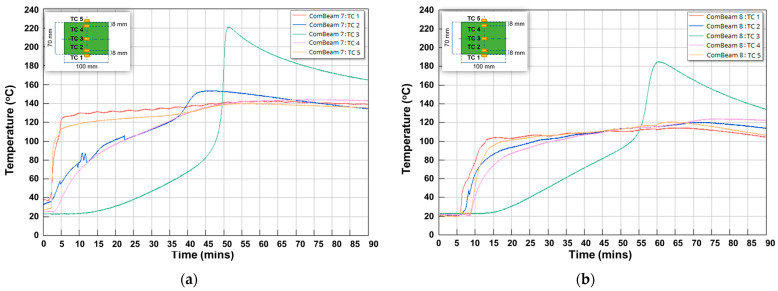
Detailed thermocouple measurements during the curing process for (**a**) ComBeam 7 with the mold heating system set to 130 °C oil temperature and (**b**) ComBeam 8 with the mold heating system set to 110 °C oil temperature.

A detailed investigation into the thermal changes during the curing process of ComBeam 7 and 8 samples is demonstrated in Figure 14. This figure depicts temperature changes using the TCs (TC 1 to TC 5), illustrating the thermal variations occurring in the ComBeam samples over a 90-min period, illustrating the distinct thermal responses and peak temperatures characteristic on the curing behavior of each ComBeam. The extensive temperature data presented here have been carefully analyzed, shedding light on a wide range of heat transfer mechanisms involved in the cases of ComBeam 7 and 8. 

The oil temperature in the mold heating system was set to 130 °C for the curing process of ComBeam 7, considering the peak temperature of the HP-DSC results mentioned in Figure 10a. However, even with the mold heating system set to 130 °C, there is an approximate 5 °C difference between the upper and lower mold temperatures, as indicated by the curves of TC 1 and TC 5 in Figure 14a. Due to the significantly high temperature of approximately 220 °C observed from TC 3, embedded at the neutral axis of ComBeam 7, it was deemed necessary to revise the mold temperature for the subsequent sample, ComBeam 8. Consequently, the mold heating system was set to 110 °C for ComBeam 8, as illustrated in Figure 14b. This adjustment was based on the initial temperature of the cross-linking reaction obtained from the HP-DSC analysis of the OM11 prepreg system, as presented in Figure 10a. 

**Figure 15:**
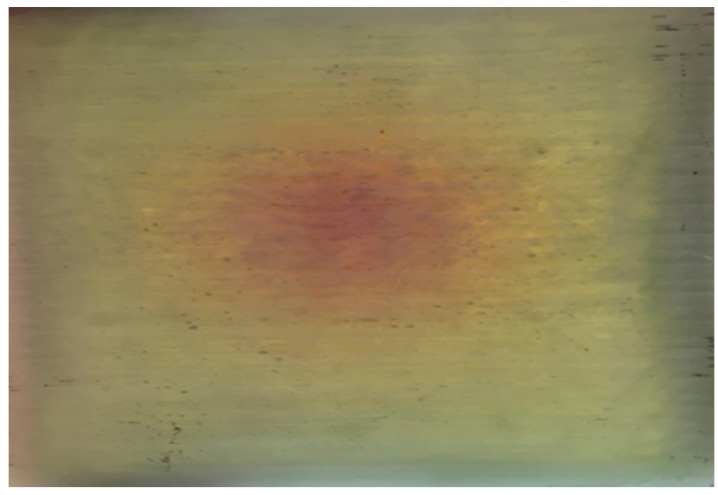
Central region of a ComBeam 7 sample cross-section: over-curing manifested by red hue intensification and subsequent resin degradation, indicative of elevated exothermic activity.

The adjustment aims to prevent overcuring and facilitate the material in reaching its optimal state for property enhancement. This aligns with research conducted by Q. Liang and colleagues, which underscores the significant role of heating rate in curing kinetics [35]. Another factor contributing to non-uniformity is illustrated in Figure 14, where differences in heating rates across various parts of the ComBeam samples are shown to impact the characteristics of the cured composite differently. Despite the calibrated settings, the maximum surface temperatures recorded via TC 1 and TC 5 exceed the intended values. This indicates a strong exothermic reaction within the resin system, causing temperatures to rise beyond the controlled mold temperature, which could significantly impact the curing behavior and the overall mechanical properties of the ComBeam samples. For ComBeam 7, surface temperatures were measured at 142.67 °C and 139.79 °C, while for ComBeam 8, surface temperatures of 120.59 °C and 114.30 °C were observed. These readings were influenced by both the mold heat and the exothermic reaction from the curing process. The internal temperatures calculated by TC 2 and TC 4, positioned 8 mm from the surface, provide valuable insight into the thermal dynamics inside the composite. ComBeam 7 exhibits higher temperatures compared to ComBeam 8, with peak internal temperatures reaching 153.40 °C and 143.98 °C, respectively. Conversely, ComBeam 8 shows slightly lower temperatures of 120.15 °C and 123.99 °C. Additionally, TC 3, tasked with monitoring core temperatures, registers maximum values of 221.5 °C for ComBeam 7 and 184.82 °C for ComBeam 8. These elevated core temperatures result from exothermic reactions occurring at the core of the composite during the curing process, as further depicted in the cross-sectional view of Figure 15, illustrating overcure and resin degradation. At these high temperatures, deeper layers of the composite structure experience reduced heat dissipation due to lower thermal conductivity. Consequently, heat accumulates in the core, intensifying the exothermic reaction and leading to elevated temperatures. This phenomenon underscores the importance of effectively managing heat during curing to achieve uniform curing without risking thermal degradation.

Temperature measurements highlight the importance of precise thermal management during curing to prevent material degradation and ensure composite integrity. Lower mold temperatures for ComBeam 8 reduce core overheating but extend the cycle time by ten minutes, showing a trade-off between thermal control and manufacturing efficiency. The TC data, combined with FBG sensor data mapping strain and temperature change, provide a comprehensive understanding of material behavior under thermal stress. This dual-sensor approach correlates internal temperature with strain, identifying high thermal stress points that may cause deformation or structural weaknesses. By improving predictability and control of the curing process, this strategy advances composite manufacturing, ensuring integrity and stability.

### 3.2. Investigation of Static Three-Point Bending Results of Composite Beams with Finite Element Analysis Correlation

**Figure 16:**
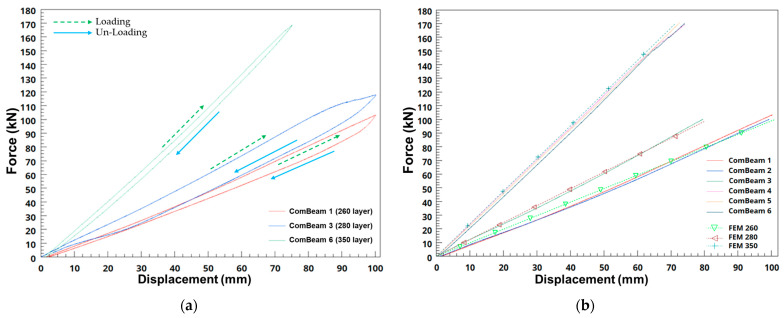
Force-displacement results; (**a**) Force-displacement curves for thick ComBeam samples and (**b**) Comparison of FE simulations with experimental stiffness measurements under specified loading conditions.

The results of the static three-point bending tests conducted on a subset of laminated ComBeams are presented in this study. Two out of six samples tested were equipped with embedded FBG sensors and surface-mounted SGs. This section also discusses the calibration studies of the FBG sensors and leads to the measurement of internal strains, providing critical insights. A comprehensive approach was adopted to integrate calibrated sensor data with FEA to enhance the understanding of material behavior under bending stresses. In particular, the stiffness of each ComBeam was rigorously evaluated, considering its internal strain distribution, to ensure a thorough assessment of stiffness and structural integrity under various stress conditions. The static three-point bending test was conducted on each ComBeam sample as shown in Figure 7. The force-displacement results of the static three-point bending test from the MTS servo hydraulic actuator are depicted in Figure 16a for each thickness of the ComBeam samples. This test, structured as a displacement-controlled, low-frequency assessment, primarily aims to measure stiffness, calibrate the FBG sensors, and record strain using both the FBG sensors and SGs at designated sensor locations, as outlined in Figure 3. Such a systematic approach is crucial for gathering accurate and comprehensive data essential for a detailed evaluation of the mechanical properties and structural integrity of the ComBeam samples, and for correlating these findings with FEM predictions using GNA. The force-displacement relationships obtained from the FEM simulations closely align with the results from the static three-point bending tests, as illustrated in Figure 16b. This strong correlation indicates the accuracy of the FEM in predicting the mechanical behavior of the ComBeam samples under load. Additionally, stiffness metrics, crucial for assessing structural integrity, are comprehensively summarized. These metrics include outcomes from the FEAs, where LD effects were considered to accurately model material behavior under varying stress levels. This integration significantly enhances the predictive accuracy of FEM models, bridging the gap between theoretical simulations and empirical physical testing results.

Therefore, in ComBeams, the importance of LD effects under FE analysis is highlighted when compared to the empirical data in Table 5. Including LD effects is crucial for accurately modeling LD modes, ensuring the model’s enhanced accuracy, especially under high load conditions. Table 5 shows that FEA with LD effects predicts experimental stiffness values for thicker samples more accurately. For instance, for a thickness of 70 mm, the GNA predicts a stiffness of 2373.3 N/mm, while the non-LD model predicts 2386.1 N/mm, compared to the experimental measurement of 2326.8 N/mm. The GNA model is accurate within −2%, demonstrating its ability to account for nonlinearities, unlike the non-LD model’s −3% deviation.

However, the FE analyses revealed intriguing discrepancies in thinner samples, such as the ‘Coupon Sample’ with a thickness of 5.2 mm. In this case, the LD analysis predicted a stiffness of 714.3 N/mm, deviating by −6% from the experimental result of 675.1 N/mm, compared to a closer −3% deviation by the non-LD analysis. This deviation indicates that while LD analysis substantially improves accuracy for thicker samples by effectively addressing LD modes, its application in thinner samples may not always achieve the same level of accuracy.

The empirical alignment between simulated and actual test results underscores the importance of incorporating LD effects. The data indicate that the significance of LD increases with the structural complexity of the material, denoted by its thickness. This is evident in the contrast of stiffness values between LD and non-LD analyses, especially as the samples become thicker. For instance, ComBeam 5 and ComBeam 6 consistently show a −1% deviation in favor of the LD analysis when compared to the experimental results, highlighting the superior accuracy of LD analysis in these cases.

**Figure 17:**
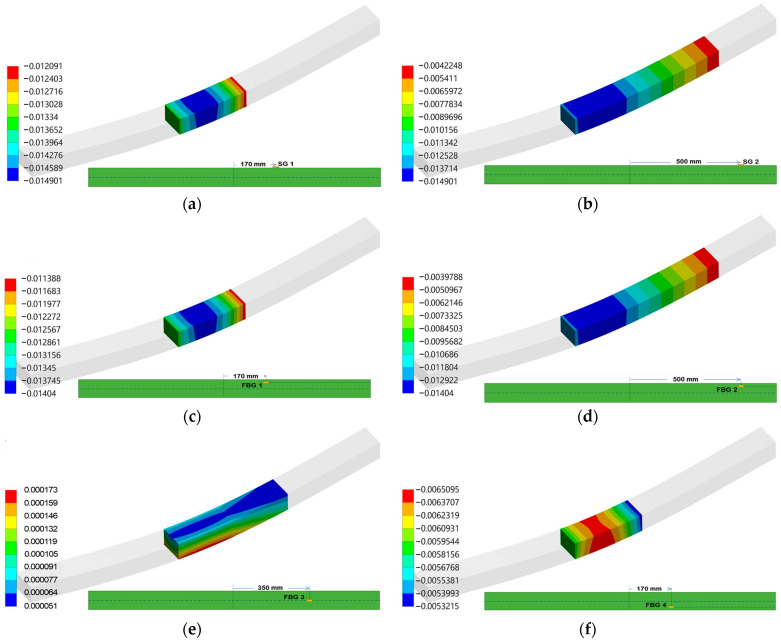

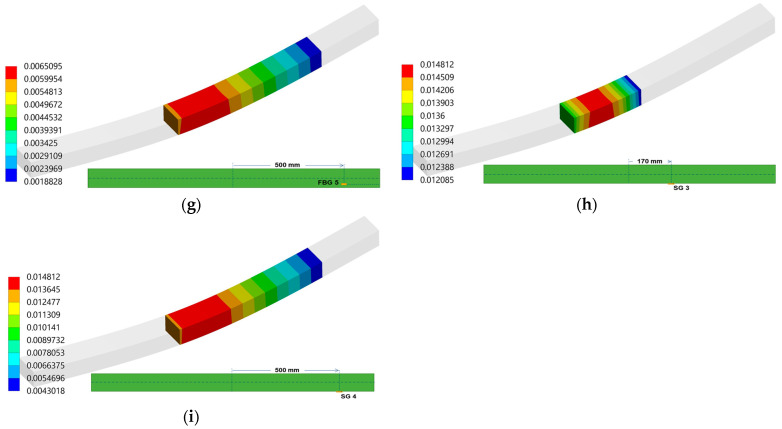
Geometrically Nonlinear Analysis under 170 kN load via FEM350 model depicting strain distribution at FBG sensor and SG points of experimental setup. The inset diagrams detail the placement of each sensor point within the ComBeam 5 & 6 as: (**a**) SG 1, (**b**) SG 2, (**c**) FBG 1, (**d**) FBG 2, (**e**) FBG 3, (**f**) FBG 4, (**g**) FBG 5, (**h**) SG 3, and (**i**) SG 4.

In summary, the integration of LD effects in FEAs aligns with theoretical expectations of improved accuracy in modeling LD modes. This integration represents a significant advancement in the predictive modeling of composite materials under actual loading conditions. The strain data from the static three-point bending tests on ComBeam 5 and 6, along with the strain projections derived using the FEM350 model (Figure 17), are presented in Table 6. The wavelength data captured by the FBG sensors during the tests were converted to strain values using Equation (1) with the Ncode 2023 software. This procedure ensures the assessment of structural integrity and strain response, demonstrating consistency between samples and validating the experimental method.

However, discrepancies observed during the tests, coupled with variations in the manufacturing of the composite material, may cause alignment errors in the produced FBG sensors, necessitating further investigation. The literature indicates that the accuracy of FBG sensors can be compromised by the formation of resin pockets around embedded fibers or shifts in fiber orientation during production. Improved embedding methods and stringent quality control are essential to achieving accurate measurements [38,39].

Additionally, deviations in strain measurements can arise from material property dispersion across the beam cross-sections. Inhomogeneities resulting from uneven temperature and pressure distributions during processing may shift the neutral axis, creating strain distributions that differ from those assumed in the FE model [40,41]. Addressing these issues in the future will enhance the accuracy and practicality of sensors for critical strain measurements.

**Figure 18:**
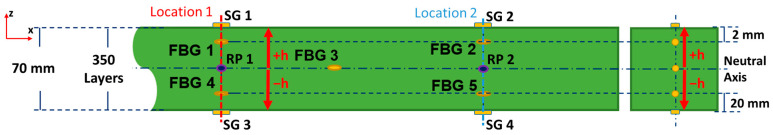
The schematic illustrates the calibration configuration of ComBeam samples, detailing the arrangement of SGs and FBG sensors across a cross-section aligned orthogonally to the neutral axis.

The FBG sensor data were meticulously calibrated against readings from the surface mounted SGs and the FE results listed in Table 6 to correct any potential misalignments of the FBG sensors within the composite structures. Figure 18 illustrates the schematic of the instrumented ComBeam samples, showing embedded FBG sensors at two critical locations. The cross-sectional dimensions of the beam and the precise locations of the FBG sensors and SGs relative to the compression (+h) and tension (−h) regions are also depicted in Figure 18. The FBG 1 and FBG 4 sensors are aligned at Location 1, calibrated with SG 1 and SG 3, respectively; similarly, the FBG 2 and FBG 5 sensors at Location 2 are calibrated with SG 2 and SG 4. All sensors are positioned on the same cross-section, perpendicular to the neutral axis.

Strategic placement of the SGs, in proximity to the FBG sensors, ensures the accuracy of the strain correlation. Reference Points (RPs) 1 and 2 designate specific positions on the neutral axis critical for determining the CC of sensor FBG 3. The CC at RP 1 is computed as the mean of the CCs for the FBG 1 and FBG 4 sensors. Conversely, the CC at RP 2 is derived from the CCs of the FBG 2 and FBG 5 sensors. Subsequently, the CC for sensor FBG 3 is calculated by averaging the CCs obtained at RPs 1 and 2.

Once calibrated, the sensor data enables an accurate depiction of the strain variation across the cross-section of the beam, denoted as ${Ɛ}_{xx}$(z), which can be described by the following linear relationship:

(2) $${Ɛ}_{xx}(z)=a_{0}+a_{11}.z$$

where z is the distance from the neutral axis, and $a_{0}$ and $a_{11}$ are the coefficients determined from the strain readings at the extreme fibers of the cross-section:

(3) z=±h

(4) $${Ɛ}_{xx}^{+}\;=\;a_{0}+a_{1}.(+h)$$

(5) $${Ɛ}_{xx}^{-}\;=\;a_{0}+a_{1}.(-h)$$

The coefficients were calculated using the strains measured at the top surface ${Ɛ}_{xx}^{+}$ and bottom surface ${Ɛ}_{xx}^{-}$ as follows:

(6) $$a_{0}=\frac{{Ɛ}_{xx}^{+}+{Ɛ}_{xx}^{-}}{2}$$

(7) $$a_{1}=\frac{{Ɛ}_{xx}^{+}+{Ɛ}_{xx}^{-}}{2h}$$

Applying these coefficients, the strain profiles for Location 1 and Location 2 were computed as follows:

(8) $${Ɛ}_{xx}\left(z)=\frac{{Ɛ}_{SG\;1}+{Ɛ}_{SG\;3}}{2}+\frac{{Ɛ}_{SG\;1}-{Ɛ}_{SG\;3}}{2h}.z\;(\mathrm{Location}\;1\right)$$

(9) $${Ɛ}_{xx}\left(z)=\frac{{Ɛ}_{SG\;2}+{Ɛ}_{SG\;4}}{2}+\frac{{Ɛ}_{SG\;2}-{Ɛ}_{SG\;4}}{2h}.z\;(\mathrm{Location}\;2\right)$$

Table 7 presents a crucial dataset for evaluating the performance of FBG sensors in the field of SHM. It compares the strains measured by the FBG sensors in the ComBeams with those calculated from the surface-mounted SGs under a static 170 kN load. This comparison serves to validate the linear strain distribution model and ensure the reliability of the FBG sensors before their application in dynamic or fatigue testing scenarios under controlled environments. The calculated strains via surface-mounted strain SGs, derived from Equations (8) and (9), serve as a theoretical baseline against which the FBG measurements were evaluated. Discrepancies between these datasets are crucial for understanding the real-world applicability of the linear strain distribution model. It was observed that the strains measured by the FBG sensors exhibit variance when compared to the SGs calculations, necessitating the use CCs. These coefficients are crucial for adjusting the raw FBG data to align with the expected strain values, thereby ensuring the precision of structural assessments.

As studied in the literature, the angular relation between the sensor axes is critical; an optimal angle maximizes accuracy, and any deviation can introduce errors in the captured strain information. This sensitivity to orientation can significantly influence the readings of FBG sensors, necessitating precise calibration to ensure that tangential strain components are accurately isolated and the effects of radial strains, which may act as noise, are minimized [38]. 

Embedding FBG sensors in composite structures significantly impacts the effective measurement of strains due to the complex strain field surrounding the sensors. These sensors are passive and integral components of the mechanical dynamics within the composite matrix, experiencing effects such as stress concentration and variations in material properties [39]. This intricate interplay with the environment necessitates a robust calibration protocol to accurately measure strain.

The detailed CCs calculated influence the post-processing of the raw data collected by the FBG sensors to ensure that the measured strain values closely match those from surface mounted SGs and the FE model theoretical predictions presented in Table 8. This calibration process, essential for mitigating the effects of external influences such as temperature changes and mechanical stress during the embedding process, requires a comprehensive approach for proper SHM. The calibration strategy, developed through in-depth analysis and empirical data, including the strain results from the FE analysis, represents a systematic iteration to improve sensor accuracy through methodical changes and subsequent validations [25,26,27]. Advancements in structural engineering rely on this diligent approach for reliable strain measurements, as it provides an effective framework for using FBG sensors in monitoring the integrity of composite structures.

Comparing the data from the FBG sensors with the FEA presented in Table 8, it becomes evident that the calibration of the FBG sensors increasingly aligns with the FE predictions in Figure 17 when calibrated experimental results are considered. However, there remains a notable deviation for the sensor located on the neutral axis. This deviation can be attributed to the FE analysis’s assumption of uniform material properties, which fails to account for variations introduced by manufacturing processes, particularly exothermic reactions. Such variations significantly impact sensor accuracy, as discussed previously in Section 3.1, presenting a different perspective from empirical data to theoretical models.

This underscores the importance of recognizing manufacturing-induced material inconsistencies in both sensor calibration and FE analysis, highlighting the need for refined modeling approaches that consider the intricate realities of material behavior. Variations in material properties across the cross-section also contribute to the observed strain deviations [39]. These variations can lead to shifts in the material’s neutral axis, thereby invalidating the assumptions of a linear strain distribution model. Such shifts are influenced by factors such as the thermal conductivity of the composite, the autocatalytic nature of curing reactions, and parameters of the curing process.

Furthermore, stress concentrations affecting FBG sensors situated between different layers within the cross-section of composite samples vary due to distinct pressure and temperature effects on each embedded FBG sensor during the manufacturing process. These complexities underscore the significance of accounting for such variables to ensure precise strain measurements and underscore the necessity for comprehensive approaches in material characterization and sensor calibration [38,40]. Variations in FBG sensor readings indicate orientation and material property discrepancies within the composite cross-section, which are critical for accurate strain measurements and for validating experimental values against Finite Element model predictions. The data presented in Table 8 illustrate how various factors interact to influence FBG sensor accuracy in SHM. Discrepancies in strain readings highlight the need to consider sensor orientation and material property variations. Understanding these impacts aids in the development of improved calibration techniques and advanced SHM methodologies, thereby enhancing predictive modeling and engineering practices for precise and reliable structural assessments.

### 3.3. Dynamic Three-Point Bending Tests on Composite Beams with Integrated Health Monitoring 

Based on insights from the CCs acquired during static evaluations, this study explored the dynamic fatigue behaviors of ComBeam 5 and ComBeam 6 under cycling fatigue test conditions. The TC measurements indicate negligible temperature generation on the surface during 1 Hz fatigue tests with 35 mm displacements, allowing the use of the FBG sensors to monitor the mechanical properties of ComBeam specimens without thermal noise interference. In dynamic tests, the early failure of surface-mounted SGs around 10,000 cycles underscores the robustness and reliability of FBG sensors, which functioned seamlessly up to 158,000 cycles in the testing of ComBeam 5. This test concluded successfully, exceeding the strength benchmark established by the leaf spring and demonstrating the performance of FBG sensors for continuous fatigue monitoring.

**Figure 19:**
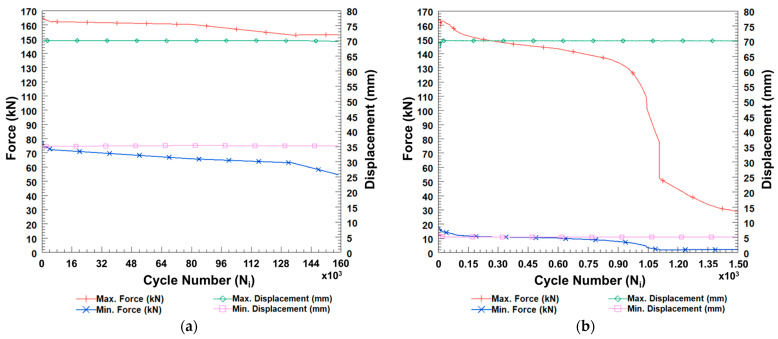
Graphical data from three-point bending fatigue tests on (**a**) ComBeam 5 and (**b**) ComBeam 6.

Figure 19a,b illustrates the force-displacement measurements as a function of cycle number for ComBeam 5 and ComBeam 6, respectively. These graphical representations are essential for scrutinizing fatigue behavior of the ComBeams, indicating the capabilities of the integrated SHM system. 

The fatigue behavior of PMCs can be divided into three distinct stages, which is consistent with earlier studies [24,25,26,27]. These stages, as described in the literature, align well with the characteristic responses of such materials under cyclic loading, as illustrated in Figure 19.

The first phase is characterized by a pronounced non-linear decrease in stiffness, primarily driven by the rapid development of matrix cracks. This typically occurs within the initial 15–25% of the fatigue life [25]. This stage, depicted in Figure 19a, corresponds to the initial cycles during which ComBeam 5 experiences a decline in both maximum and minimum forces, likely due to factors such as matrix cracking, residual curing stresses, and voids or inconsistencies in the composite material.

The subsequent phase exhibits a more gradual, linear decline in stiffness, spanning approximately 15–20% to 90% of the fatigue life [25]. This reduction is attributed to continued matrix cracking, fiber debonding, and delamination, as observed in Figure 19a throughout most of the cyclic loading duration.

In the terminal phase, occurring within the final 5–10% of the fatigue life, there is a sharp, non-linear decline in stiffness attributed to significant fiber fractures and the accumulation of critical damage. This phenomenon was not observed in ComBeam 5 [25].

**Figure 20:**
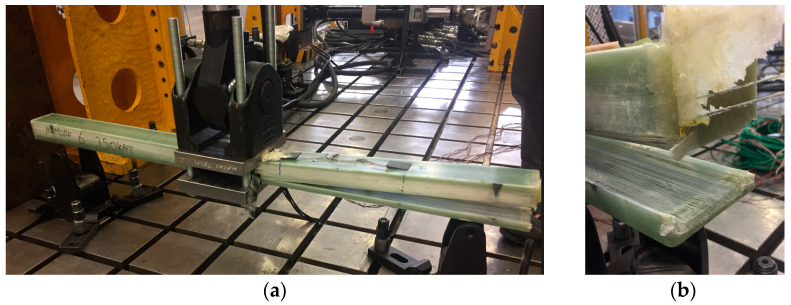
Post-failure investigation of ComBeam 6 following the three-point fatigue testing. (**a**) The images display the ComBeam 6 failure, (**b**) illustrating the fracture location and its pattern.

In contrast, the fatigue characteristics of ComBeam 6, illustrated in Figure 19b, enter this phase after only 1107 cycles. This highlights that larger displacement amplitudes, even at a lower frequency of 0.5 Hz, notably accelerate the onset of critical damage. This rapid progression to failure, as seen in Figure 20, contrasts with ComBeam 5, which successfully endures the entire range of tested cycles without failure.

Scholarly research consistently indicates that higher displacement amplitudes during cyclic loading expedite the transition to the critical damage phase, thereby diminishing the overall fatigue life of composite materials. This accelerated damage progression, which includes matrix cracking, fiber-matrix debonding, and eventual fiber fracture, is attributed to the intensified displacement conditions [26].

**Figure 21:**
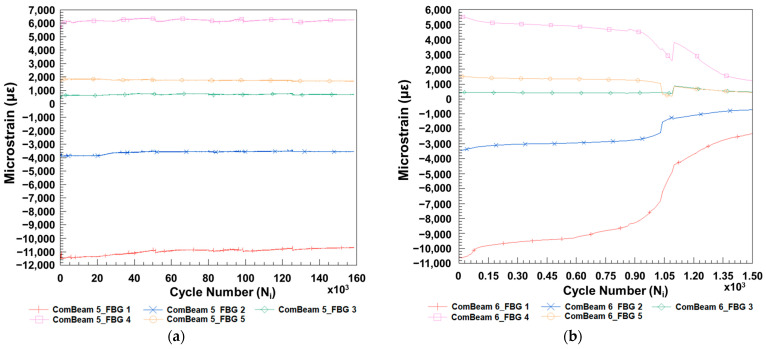
Microstrain response of FBG sensors embedded in (**a**) ComBeam 5 and (**b**) ComBeam 6.

The experimental strain data collected by the FBG sensors during the fatigue tests conducted on ComBeam 5 and ComBeam 6, given in Figure 21a,b, respectively, provide a comprehensive comparative analysis of fatigue responses in composite materials. These figures illustrate the variations in maximum strain values, which are crucial for understanding the material’s behavior under cyclic loading. Namely, recalling that the fatigue test is conducted under constant strain conditions, it should be expected that the maximum and minimum values of the FBG strain obtained experimentally would also be constant. As can be seen from these figures, the FBG strain values change as a function of the number of cycles, and these changes exhibit a three-region trend similar to the force-cycle number graphs in Figure 19. This indicates that by monitoring the strain changes due to localized damage under fatigue loads in the material, the remaining useful life of the composite beam structure in terms of fatigue cycles can be predicted [27]. Essentially, the FBG sensors offer insights into internal strain dynamics that external measurements based on displacement alone may overlook. While ComBeam 6 experiences all three stages of the fatigue response, leading to failure within a relatively short testing period as seen in Figure 20, ComBeam 5, due to its longer test duration, does not fail and therefore does not distinctly exhibit the three fatigue phases. The observed variation in strain serves as a crucial indicator of the structural integrity and durability of the composite beams under repetitive loading conditions. These data provide invaluable insights for predicting the service life and formulating maintenance strategies for composite structures across various engineering applications.

**Figure 22:**
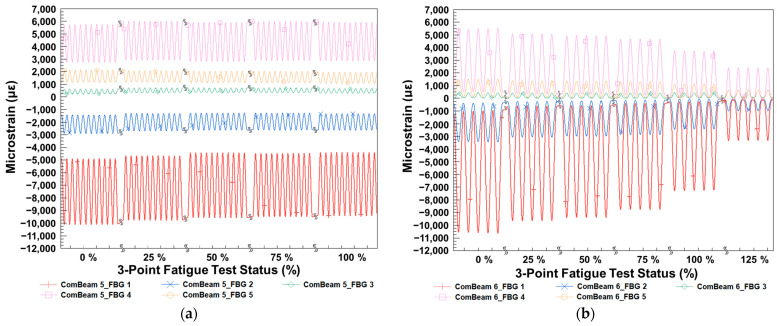
FBG strain measurement data captured at quarter-based percentages of the fatigue tests for (**a**) ComBeam 5 and (**b**) ComBeam 6.

Figure 22 compares the microstrain amplitudes of ComBeam 5 and ComBeam 6 throughout the fatigue tests, presenting data in quarter-based intervals of the total test duration. Figure 22a shows continuous strain behavior for ComBeam 5 under a 35 mm displacement amplitude, while Figure 22b illustrates the same for ComBeam 6 under a 65 mm displacement amplitude. The color-coded representation delineates the varied microstrain amplitudes measured by each FBG sensor, indicating the strain distribution across the beams subjected to three-point fatigue testing.

Figure 22a demonstrates the consistent cyclic microstrain levels of ComBeam 5, highlighting its robust material performance under repetitive loading conditions. The stability in strain amplitudes across all testing quarters indicates sustained structural integrity, with no signs of catastrophic failure within the test parameters.

In contrast, Figure 22b depicts notably varying strain field as a function of cycle number during the testing of ComBeam 6. It shows a trend of decreasing maximum strain values as the test progresses. This decrease becomes particularly pronounced after reaching the 75% mark of the fatigue test, followed by a sharp decline that leads to material failure after exceeding the 100% fatigue threshold. At the 125% fatigue test status, a significant reduction in strain is observed, indicating the post-failure state of ComBeam 6, as corroborated by Figure 20b. This reduction in strain after failure suggests the loss of material resilience and the breakdown of structural coherence, consistent with the final phase of fatigue life as postulated by theoretical failure models [24,25,26,27]. The data from ComBeam 6 exhibit all characteristics leading up to and beyond the failure point, crucial for understanding the total fatigue behavior of the material.

The readings from FBG sensors can explicitly track the transition from one phase to another in the fatigue life, providing quantifiable and predictive insights valuable for predicting the end-of-service life for such materials in real-world applications. The reduced strain levels and failures demonstrated in ComBeam 6 correlate well with theoretical models, emphasizing the importance of monitoring composite materials. Thus, FBG sensors show potential as a crucial component in SHM systems.

The data presented in Table 9 supplement the strain behavior observed in Figure 22, facilitating a detailed quantitative assessment of the microstrain experienced by ComBeam 5 and ComBeam 6 during the three-point fatigue test. The table identifies the amplitude of microstrain at each condition, providing quantitative data for comparing the strain experienced by the sensors at critical locations throughout the fatigue test. ComBeam 5 shows relatively stable strain values and does not fail even at the 100% fatigue test status, indicating its ability to withstand repetitive loading without structural failure. This consistency in strain amplitude across different test intervals affirms the mechanical integrity observed in Figure 22a, further supporting ComBeam 5’s endurance throughout the testing duration. 

The strain response data for ComBeam 6, as outlined in Table 9, demonstrate a dynamic progression, initially characterized by a reduction in absolute microstrain amplitudes. This reduction signifies the presence of internal structural defects within the composite material. As fatigue loading persists, the material exhibits a significant decrease in strain amplitude upon reaching structural failure, as illustrated in Figure 20. The sequence, detailed in the table, elucidates the gradual degradation of the composite under cyclic loading, culminating in a substantial inability to sustain strain, particularly evident in the 125% post-failure strain values. This trajectory corresponds with the insights from Figure 22b, where a notable decrease in strain post-failure is observed, indicating material failure.

The observations from Table 9 confirm the efficacy of FBG sensors as diagnostic tools in real-time SHM, validating their effectiveness in capturing critical data that reflect the condition of the material during fatigue testing. The correlation between the strain data from the FBG sensors and the physical evidence of material behavior reinforces the reliability of these sensors in predicting material performance and lifespan in engineered systems. The degradation pattern leading to the ultimate failure of ComBeam 6 aligns with established fatigue failure stages and models discussed in the literature and exemplified by the analyses in Figure 22.

## 4. Conclusions

This study represents a significant advancement in SHM for composite structures, detailing the lifespan of large-scale laminated ComBeams from manufacturing through virtual modeling to physical testing. By integrating FBG sensors and SGs, this research achieved precise monitoring of residual strains and optimized the curing process through temperature adjustments informed by the TC data. This dual monitoring technique improves material exotherm and uniformity during curing and enables real-time SHM during static and dynamic (fatigue) loading conditions.

The calibrated strains, combined with the SG data obtained from the FBG sensors, demonstrate the synergy between advanced sensor technologies and conventional experimental methods, enhancing the accuracy and reliability of SHM techniques. The use of GNA to incorporate LD effects within the FE model improved the correlation between numerical and experimental data, allowing a detailed analysis of stiffness properties under various conditions, particularly for thicker samples.

The research highlights the capability of FBG sensors to detect early material degradation, outperforming SG sensors, which fail prematurely under the same stresses. Detailed analysis of the laminated beam in a three-point bending test reveals significant findings about the load-carrying behavior of ComBeams. The results show that when FBG sensors are used, there is a notable deviation of the neutral axis compared to the FE models, which was attributed to non-uniform material properties resulting from the exothermic reaction during curing. This insight, combined with force-displacement data from displacement-controlled experiments, elucidates the complex relationship between composite material composition, manufacturing processes, and the structural efficiency of laminated composite beams.

Comparing experimental results with stiffness values obtained from the FE model validates the model used in this study and underscores the methodology’s potential for understanding the intricate behavior of composite materials. This approach, which combines theoretical models with empirical data, emphasizes the complex interdependencies defining the performance of composite structures and sets a direction for future material science and engineering research.

In conclusion, this research significantly contributes to materials science and engineering, offering valuable practical applications in SHM. It underscores the importance of combining sensor technology with numerical modeling to predict the behavior of complex composite structures. Further development of these techniques will provide researchers with enhanced tools to address the highly heterogeneous nature of composite materials, ensuring safer and more reliable composite structures in various industrial applications. This holistic study exemplifies the benefits of integrating experimental and numerical strategies to advance SHM.

Future research will focus on composite leaf springs of HCVs with varying cross-sectional thickness along their length. Additionally, integrating TCs with FBG sensors in thick ComBeams will be essential for monitoring strain distribution during curing. This integration aims to separate thermal effects on FBG sensors to understand pressure distribution during manufacturing. Furthermore, material characterization across the cross-section of thick composite structures will be necessary to identify non-uniform thermal effects on material properties. These efforts will address the challenges observed in this study and advance the development of robust, reliable thick composite components for structural applications. Moreover, force-controlled conditions are to be considered for fatigue monitoring, where the applied load remains constant during the measurement of the resulting strain, allowing direct observation of the material’s response to stress. This approach might be particularly beneficial for tracking damage evolution in real-time, as it can elucidate how the material behaves under specific loading.

## Figures and Tables

**Table 1:** An overview of fabricated laminated ComBeam samples: number of prepreg layers and geometric specifications (length, width, and thickness).

Sample Name	Total Number of Prepreg Layers	Dimensions (Length × Width × Thickness) (mm)
ComBeam 1	260	1600 × 100 × 52
ComBeam 2	260	1600 × 100 × 52
ComBeam 3	280	1600 × 100 × 56
ComBeam 4	350	1600 × 100 × 70
ComBeam 5	350	1600 × 100 × 70
ComBeam 6	350	1600 × 100 × 70
ComBeam 7	350	1600 × 100 × 70
ComBeam 8	350	1600 × 100 × 70

**Table 2:** Material properties of 300 GSM OM11 prepreg obtained from coupon samples.

	Material Property	Experimental Result	Material Property	Experimental Result
Prepreg	E_x_ (GPa)	43.3	(σ_x_)_T_ (MPa)	881.35
	E_y_ (GPa)	10.91	(σ_y_)_T_ (MPa)	51.54
	E_z_ (GPa)	10.91	(σ_z_)_T_ (MPa)	51.54
	ν_xy_	0.12	(σ_x_)_C_ (MPa)	−637.34
	ν_yz_	0.12	(σ_y_)_C_ (MPa)	−176.74
	ν_xz_	0.31	(σ_z_)_C_ (MPa)	−174.74
	G_xy_ (GPa)	5.07	τ_x_ (MPa)	71.05
	G_yz_ (GPa)	4.23	τ_y_ (MPa)	45.71
	G_xz_ (GPa)	5.07	τ_z_ (MPa)	71.05
Resin	ρ (kg/m^3^)	1.116	ν	0.35
	E (GPa)	3.780	τ (MPa)	1400

**Table 3:** List of samples with their prepreg layer count, dimensions, and a column for mesh elements, detailing variations in structure for the FEA.

FEM Code	Sample Name	Total Number of Prepreg Layers	Dimensions (mm) (Length × Width × Thickness)
FEM 26	Coupon Sample	26	100 × 15.26 × 5.2
FEM 260	ComBeam 1, 2	260	1600 × 100 × 52
FEM 280	ComBeam 3	280	1600 × 100 × 56
FEM 350	ComBeam 4, 5, 6	350	1600 × 100 × 70

**Table 4:** Residual strain measurements for ComBeam 5 and ComBeam 6 samples using FBG Sensors.

Sample Name	FBG #	Residual Strain after Curing (με)	Calibrated Residual Strain after Curing (με)	Residual Strain after Post-Curing (με)	Calibrated Residual Strain after Post-Curing (με)	Post-Cure Calibrated Strain Change %
ComBeam 5	FBG 1	−560	−636	−441	−501	21%
	FBG 2	−469	−631	−383	−515	18%
	FBG 3	−798	−836	−746	−782	7%
	FBG 4	−537	−566	−499	−526	7%
ComBeam 6	FBG 1	−577	−561	−500	−487	13%
	FBG 2	−749	−782	−679	−708	9%
	FBG 3	−967	−911	−876	−825	9%
	FBG 4	−591	−578	−482	−472	18%

**Table 5:** Comparison of stiffness measurements in ComBeam samples: empirical test results vs. FEA with and without LD.

Sample Name	Experimental Result of Stiffness (N/mm)	FEM Stiffness (Non-LD) (N/mm)	FEM Stiffness (LD) (N/mm)	Test Results vs. FEM (NonLD) (%)	Test Results vs. FEM (LD) (%)
Coupon	675.1	693.2	714.3	−3%	−6%
ComBeam 1	1054.3	989.2	1039.0	6%	1%
ComBeam 2	1017.3	989.2	1039.0	3%	−2%
ComBeam 3	1251.7	1232.8	1263.7	2%	−1%
ComBeam 4	2326.8	2386.1	2373.3	−3%	−2%
ComBeam 5	2351.6	2386.1	2373.3	−1%	−1%
ComBeam 6	2348.7	2386.1	2373.3	−2%	−1%

**Table 6:** In-depth strain measurement data acquired under a 170 kN load in static three-point bending tests for ComBeam 5 and ComBeam 6 utilizing surface-mounted SGs and embedded FBG sensors paired with strain projections from corresponding sensor points derived from the FEM350 FE model analysis.

Sensor Code	Measured Strain of ComBeam 5 (με)	Measured Strain of ComBeam 6 (με)	Strain Obtained by FEM 350 with LD (με)	Strain Deviation (%) for ComBeam 5: Test vs. FE (LD)	Strain Deviation (%) for ComBeam 6: Test vs. FE (LD)
SG 1	−12,347.04	−12,244.74	−12,091.00	1%	0%
SG 2	−4166.13	−3927.65	−4224.80	−1%	−7%
SG 3	13,101.35	13,055.75	12,144.00	7%	7%
SG 4	4093.95	3828.28	4301.80	−5%	−12%
FBG 1	−10,227.64	−11,836.98	−11,388.00	−11%	4%
FBG 2	−2922.20	−3551.57	−3978.80	−33%	−13%
FBG 3	457.42	473.22	50.62	87%	91%
FBG 4	5532.65	5954.29	5321.50	4%	11%
FBG 5	1999.80	1905.37	1882.80	6%	1%

**Table 7:** Strain measurement and calculation comparison for ComBeam 5 and ComBeam 6 under a 170 kN Load.

Sensor Code	Strains Measured by FBGs: ComBeam 5 (με)	Strains Calculated Via SGs: ComBeam 5 (με)	Calibration Coefficients: ComBeam 5	Strains Measured by FBGs: ComBeam 6 (με)	Strains Calculated Via SGs: ComBeam 6 (με)	Calibration Coefficients: ComBeam 6
FBG 1	−10,227	−11,619	1.14	−11,836	−11,521	0.97
FBG 2	−2922	−3930	1.35	−3551	−3706	1.04
FBG 3	457	479 *	1.05 *	473	449 *	0.95 *
FBG 4	5532	5830	1.05	5954	5827	0.98
FBG 5	1999	1733	0.87	1905	1612	0.85

* Calculations are performed using RP 1 and 2, which correspond to Locations 1 and 2, respectively.

**Table 8:** Comparative analysis of measured and calibrated FBG strain versus FE model predictions for ComBeam 5 and 6. The table outlines the calibrated strains captured by the FBG sensors versus those predicted by the FEM350 model with LD.

Sensor Code	Measured Strain of ComBeam 5 (με)	Measured Strain of ComBeam 6 (με)	Calibrated Strain of ComBeam 5 (με)	Calibrated Strain of ComBeam 6 (με)	Strain of FEM350 with LD (με)
FBG 1	−10,227.64	−11,836.98	−11,619.95	−11,521.87	−11,388.00
FBG 2	−2922.20	−3551.57	−3930.13	−3705.54	−3978.80
FBG 3	457.42	473.22	479.36	445.97	50.62
FBG 4	5532.65	5954.29	5830.38	5827.04	5321.50
FBG 5	1999.80	1905.37	1733.93	1625.04	1882.80

**Table 9:** Absolute strain amplitudes from FBG sensors during three-point fatigue testing for ComBeam 5 and ComBeam 6 samples.

Sample Name	Three-Point Fatigue Test Status (%)	FBG 1 Strain Amplitude (με)	FBG 2 Strain Amplitude (με)	FBG 3 Strain Amplitude (με)	FBG 4 Strain Amplitude (με)	FBG 5 Strain Amplitude (με)
ComBeam 5	0	5210	1463	377	2998	977
	25%	5096	1360	367	3059	898
	50%	5133	1361	352	3088	889
	75%	5020	1322	363	3052	874
	100% (not failed)	5004	1309	366	3044	866
ComBeam 6	0	9655	2878	414	4964	1566
	25%	8312	2415	367	4266	1341
	50%	8790	2583	400	4533	1443
	75%	5093	1524	235	2626	829
	100% (failed)	5283	1671	321	2653	926
	125%	2282	674	446	1645	592

## Data Availability

The data presented in this study are available on request from the authors of this paper.
